# C. E. Hawley, D.D.S.

**Published:** 1891-08

**Authors:** 


### C. E. HAWLEY, D.D.S.

Died, in Shreveport, La., June 9, 1891, suddenly, of paralysis of the heart, C. E. Hawley, D.D.S., in the fifty-first year of his age.

Dr. Hawley was born in Ithaca, N. Y., February 28, 1840. He was the son of the late Dr. Joel E. Hawley, a prominent physician of that place for many years. He went South when twenty-one years of age, and studied dentistry with the late Dr. Russell, of Nashville, Tenn. He then removed to Natchitoches, La., to practise, and there married a Miss Boultt. He subsequently settled in Shreveport, La., where he practised very successfully many years, and up to the time of his death. He graduated from the Dental College at Nashville, Tenn., in 1881, and was a member of the

Louisiana State Dental Society. Dr. Hawley was a brother of S. S. Hawley, D.D.S., now practising in Newark, N. J. He leaves a widow and six children.

